# Dynamically Driven Allostery in MHC Proteins: Peptide-Dependent Tuning of Class I MHC Global Flexibility

**DOI:** 10.3389/fimmu.2019.00966

**Published:** 2019-05-03

**Authors:** Cory M. Ayres, Esam T. Abualrous, Alistair Bailey, Christian Abraham, Lance M. Hellman, Steven A. Corcelli, Frank Noé, Tim Elliott, Brian M. Baker

**Affiliations:** ^1^Department of Chemistry and Biochemistry, University of Notre Dame, Notre Dame, IN, United States; ^2^Harper Cancer Research Institute, University of Notre Dame, South Bend, IN, United States; ^3^Computational Molecular Biology Group, Institute for Mathematics, Freie Universität Berlin, Berlin, Germany; ^4^Institute for Life Sciences and Centre for Cancer Immunology, University of Southampton, Southampton, United Kingdom

**Keywords:** class I MHC molecules, peptides, dynamics, motion, allostery, structure

## Abstract

T cell receptor (TCR) recognition of antigenic peptides bound and presented by class I major histocompatibility complex (MHC) proteins underlies the cytotoxic immune response to diseased cells. Crystallographic structures of TCR-peptide/MHC complexes have demonstrated how TCRs simultaneously interact with both the peptide and the MHC protein. However, it is increasingly recognized that, beyond serving as a static platform for peptide presentation, the physical properties of class I MHC proteins are tuned by different peptides in ways that are not always structurally visible. These include MHC protein motions, or dynamics, which are believed to influence interactions with a variety of MHC-binding proteins, including not only TCRs, but other activating and inhibitory receptors as well as components of the peptide loading machinery. Here, we investigated the mechanisms by which peptides tune the dynamics of the common class I MHC protein HLA-A2. By examining more than 50 lengthy molecular dynamics simulations of HLA-A2 presenting different peptides, we identified regions susceptible to dynamic tuning, including regions in the peptide binding domain as well as the distal α3 domain. Further analyses of the simulations illuminated mechanisms by which the influences of different peptides are communicated throughout the protein, and involve regions of the peptide binding groove, the β_2_-microglobulin subunit, and the α3 domain. Overall, our results demonstrate that the class I MHC protein is a highly tunable peptide sensor whose physical properties vary considerably with bound peptide. Our data provides insight into the underlying principles and suggest a role for dynamically driven allostery in the immunological function of MHC proteins.

## Introduction

T cell receptor (TCR) recognition of antigenic peptides bound and presented by class I major histocompatibility complex (MHC) proteins underlies the cellular immune response to diseased cells. Crystallographic structures of TCR-peptide/MHC complexes have demonstrated how TCRs simultaneously interact with both the peptide and the MHC protein [recently reviewed in (1)]. Accordingly, along with the peptide, amino acids within the class I MHC peptide binding domain directly impact TCR recognition. However, it is becoming increasingly recognized that, beyond serving as a static platform for peptide presentation, the properties of MHC proteins are modulated by different peptides. For example, peptides can change how the TCR interfaces with the α helices of the MHC protein, influencing their contribution to receptor binding and leading to what we have termed an “extension of antigenicity” from the peptide to the MHC (2–7).

Beyond TCRs, class I MHC proteins interact with numerous other proteins of the cellular immune system. These include components of the peptide-loading machinery, such as the chaperones tapasin and TAPBPR (8–12), as well a range of activating and inhibitory receptors, including the CD8 coreceptor (13) as well as a variety of natural killer (NK) receptors that serve as a “check” on MHC loss in infection and cancer (14). Surprisingly, some of these MHC-protein interactions show a peptide dependence, even though the protein-protein interfaces exclude the bound peptide. For example, the inhibitory Ly49C NK receptor in mice distinguishes between peptides bound to the class I MHC protein H-2K^b^, despite the fact that it binds at a location “underneath” the H-2K^b^ peptide binding groove (15, 16). Other classes of NK receptors also show peptide selectivity (17). In other cases, proteins that interact with class I MHC proteins distinguish between the presence or absence of tightly bound peptides, as seen with tapasin and TAPBPR (9, 10, 18–23).

As expansive architectural changes in class I MHC proteins with different peptides have not been observed, peptide-dependent tuning of class I MHC motional properties has been suggested as a mechanism through which peptide-selective binding of various proteins can be achieved (3–5, 20, 24–28). Indeed, experiments that assess protein motion, including fluorescence anisotropy, hydrogen/deuterium exchange, and NMR, have indicated the presence of dynamic communication from the peptide binding domain to other regions of the molecule, including the α3 domain and the non-covalently associated β_2_-microgloublin subunit (29–31). Thus, different peptides appear to tune the dynamics of not only the peptide binding domain, but regions throughout the molecule. This is consistent with the concept of dynamically driven allostery, in which motional changes in proteins triggered by ligand binding influences other binding interactions at distant sites (32, 33).

Recently, we described a library of extensive molecular dynamics simulations of 52 different nonameric peptides bound to the class I MHC protein HLA-A2 using available crystallographic structures for starting coordinates (34). In our previous work, we assessed how peptide composition influenced peptide motion within the HLA-A2 binding groove. Here, we used this simulation library to ask how different peptides influence the motion of the HLA-A2 protein. We found that, consistent with suggestions from experimental data, different peptides significantly impact the dynamics of the helices of the peptide binding groove, with the short arm of the α2 helix (also referred to as the α2-1 helix) showing particular susceptibility, with potential to alter recognition by TCRs and other receptors of the immune system that engage the peptide binding domain.

We also observed regions outside the peptide binding groove whose motional properties displayed a peptide dependence, highlighting that different peptides can alter the protein's entire energy landscape. Regions impacted include the α3 domain, which lies at the opposing end of the molecule from the peptide groove. Following experimental validation of this observation, we identified residues within the β_2_m subunit which were consistently utilized in propagating dynamics. The regions impacted overlap with binding sites for the CD8 coreceptor, tapasin, and various NK receptors, potentially contributing to a peptide-dependence to their binding.

Overall, our analysis suggests that the class I MHC molecule is a highly tunable peptide sensor whose biophysical properties vary considerably with the nature of the bound peptide. Potentially significant consequences from this tunability include an influence on TCR recognition or degeneracy, a peptide dependence to the strength or kinetics of the interactions with other activating and inhibitory receptors that bind class I MHC proteins, and an influence on how the ER-resident chaperones select peptides for eventual presentation.

## Results

### Peptides Alter HLA-A2 α1 and α2 Helix Dynamics

Multiple studies have demonstrated that different peptides can modulate class I MHC peptide binding groove motions (3–5, 24, 25, 35, 36, 40). To investigate peptide and MHC motions, we recently described a library of extensive molecular dynamics simulations of nonameric peptides bound to the class I MHC protein HLA-A2. The library consisted of 97, one microsecond simulations of different peptide/HLA-A2 complexes in explicit solvent using available crystallographic structures for starting coordinates (34). After accounting for repeats, validation simulations, and discarding unstable simulations, the final curated library consisted of 52 simulations of HLA-A2 bound to different nonameric peptides (Table S1).

**Figure 1:**
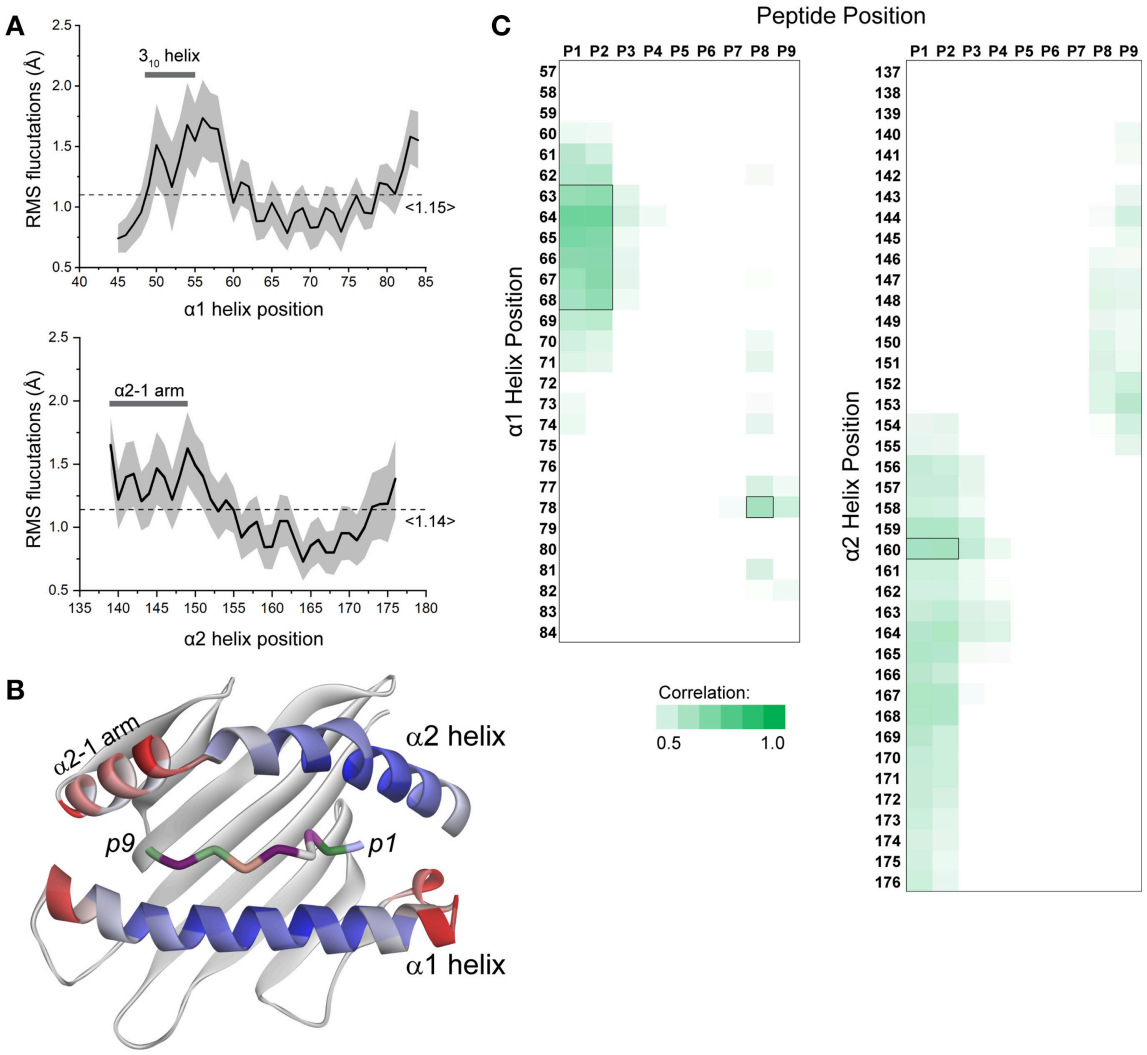
Peptide-dependent fluctuations of the HLA-A2 peptide binding groove. **(A)** RMS fluctuations were calculated for each Cα atom of the α1 and α2 helices of the HLA-A2 molecule for all 52 simulations. The solid black line represents the by-residue averages value across all simulations. The standard deviation for each residue is indicated by the gray shading. The 3_10_ portion of the α1 helix and the α2-1 arm of the α2 helix are highlighted. The overall average across each helix is shown by the dashed line. **(B)** Average fluctuation data from panel A mapped to the structure of the HLA-A2 peptide binding groove. High values are indicated in red; low values are indicated in blue (peptide amino acids are colored separately; peptide colors do not indicate fluctuations). **(C)** Correlations between peptide RMS fluctuations and HLA-A2 α1/α2 helix RMS fluctuations across all 52 simulations. Only the fluctuations of the peptide N- and C- terminal residues displayed appreciable correlations with fluctuation of the α helices, with only seven residues of the α1 helix and one residue of the α2 helix generating possessing correlation coefficients >0.6 (indicated by black boxes).

To assess whether and how peptide-dependent motions of the HLA-A2 binding groove were recapitulated by our simulations, we examined α carbon root mean square (RMS) fluctuations for every residue of the HLA-A2 α1 and α2 helices in each simulation (Figures 1A,B). These values indicate the magnitude, in Ångstroms, of the motions present for each amino acid and thus provide a snapshot of peptide dependent motion. A large range of fluctuations was observed for both helices, with the N- and C-terminal ends of each helix showing more mobility than the centers. Across the helices, the α2 helix displayed a greater variance, consistent with experimental observations showing that α2 helix dynamics are more dramatically impacted by peptide (4). The shorter arm of the α2 helix (also referred to as the α2-1 helix, adjacent to the peptide C-terminus) possessed particularly elevated mobility. Mobility or conformational variation of the α2-1 helix has been noted several times and has been implicated in influencing recognition of the peptide/MHC complexes by TCRs and other immunoreceptors, as well as playing a role in peptide loading and exchange (2, 3, 6, 9, 10, 18, 35, 36). We also observed a large variance in RMS fluctuations of the 3_10_ helical segment of the α1 helix, which much like the α2-1 helix, has been implicated as serving a function in peptide loading (27).

In studying how peptide properties were correlated with binding groove dynamics, we first asked if peptide motions were correlated with the motions of the α1 and α2 helices. We generated linear regression models between peptide and α helix RMS fluctuations and observed generally weak correlations. Only seven residues of the α1 helix and one residue of the α2 helix had correlation coefficients of 0.6 or greater with peptide positions (Figure 1C). These and other weaker correlations were restricted to regions near, and correlated with, N- and C-terminal peptide positions. There were no correlations between α helix fluctuations and the presence or absence of optimal anchor residues (i.e., leucine or methionine at position 2 and valine at position 9). Indeed, thermal stability assessments of peptide binding affinity are available for 18 of the 52 simulated complexes (37). Using these, we were unable to find any significant models that related α1 or α2 helix motions to peptide binding affinity. These results suggest that the impact of peptide on binding groove dynamics is a complex phenomenon that incorporates more than the strength of binding, as shown experimentally in previous studies (5, 38) and diagrammed in Hawse et al. (4).

### Peptides Modulate Helical Geometry Across the Binding Groove

**Figure 2:**
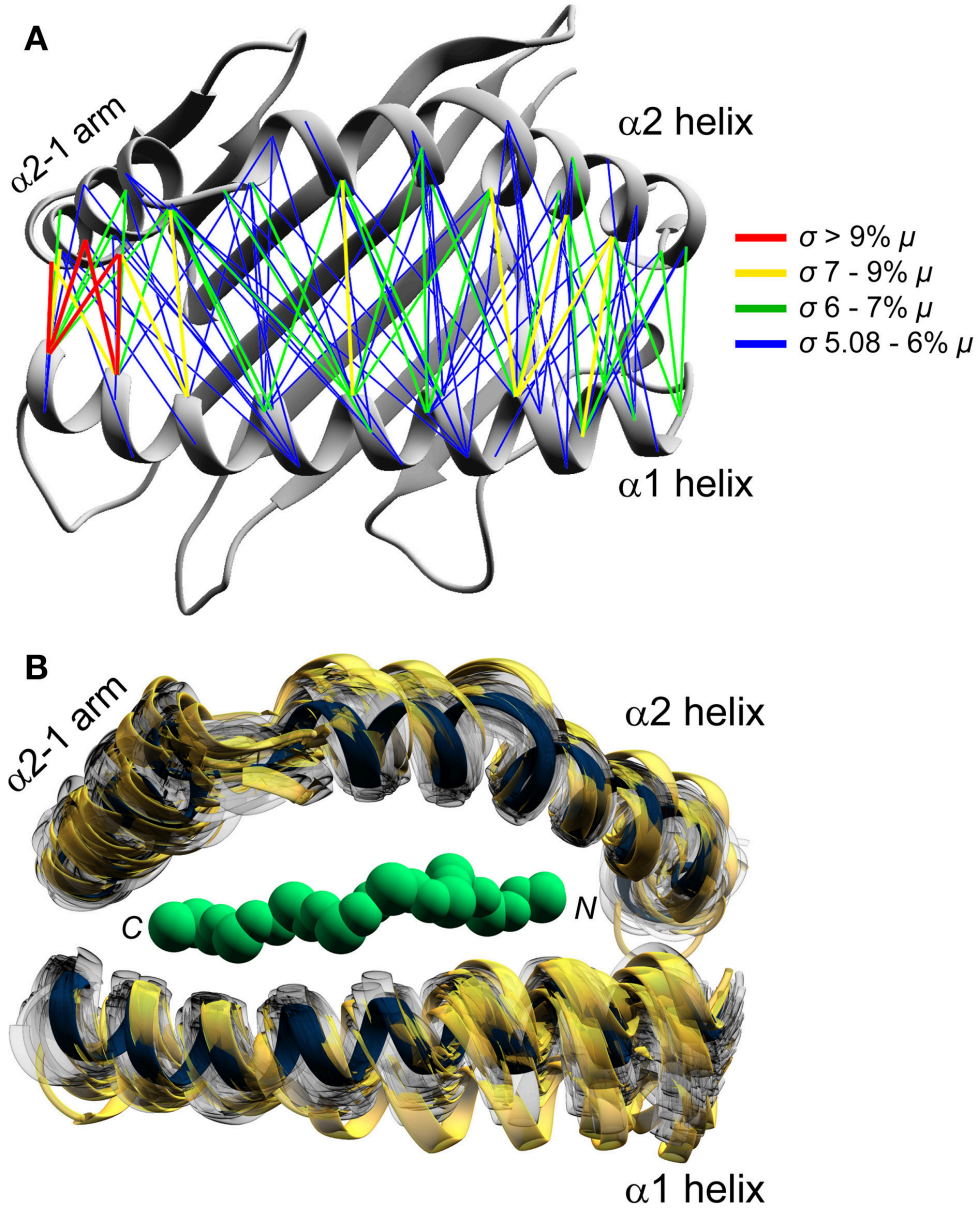
Peptide modulation of HLA-A2 binding groove geometry. **(A)** Binding groove geometry is differentially modulated by peptide, as shown by peptide dependent variances in Cα–Cα distances. Distances whose coefficients of variation were in the top 10% are shown and colored according to the inset (standard deviation [σ] greater relative to the indicated percent of the mean [μ]). The region adjacent to the C-terminal end of the peptide shows the most variation, primarily including distances involving the α2-1 helix. **(B)** 208 frames from the 52 simulations representing the least to the most divergent relative to the Tax/HLA-A2 reference structure. The reference structure is in dark blue and the 20 frames with the highest deviations are shown in gold.

We next asked how peptides alter HLA-A2 binding groove geometry. From each simulation, we determined the average distance for each pair of α carbons of the α1 and α2 helices. This resulted in an array of 1,102 pairs of distances for each simulation. From these we selected those most noticeably impacted by peptide, defined as those pairs whose coefficients of variation (i.e., the ratio of the standard deviation of the distance divided by the mean) were in the top 10%. This yielded 113 α carbon pairs whose distances were highly modulated by peptide. Rather than being focused in one region, these peptide susceptible distances were distributed across the binding groove, indicating that different peptides alter the dynamic breathing of the entire peptide binding groove (Figure 2A). The regions most susceptible to peptide, however, were the ends of the helices in proximity to the peptide C-terminus, which again included the short α2-1 helix.

To visualize the binding groove structural variations across the 52 simulations, for every 500 picoseconds of each simulation we computed α1 and α2 helix α carbon RMS deviations relative to the crystallographic structure of the HTLV-1 Tax peptide presented by HLA-A2 (39). The list of 104,000 RMS deviations ranged from 0.7 to 2.5 Å. For each simulation, we extracted the frames corresponding to the minimum and maximum RMS deviation, as well as frames representing the 33rd and 66th percentile RMS deviation. This yielded a total of four frames per simulation. These 208 frames yielded a distribution of conformations sampled during the simulations, from the least to most divergent relative to the reference structure for each simulation. The extracted structures were then mapped onto the reference (Figure 2B). Consistent with the pairwise analysis, we observed that increases in the α helix RMS deviations were associated with an overall broadening of the peptide binding groove. While we observed substantial broadening at the ends of each helix, we also observed a broadening of the central region of the binding groove, as well as substantial motions of the linker connecting the long and short arm of the α2 helix, recapitulating what has been observed experimentally.

### HLA-A2 Helix Dynamics Are Modulated by Peptide Fluctuations and Volume

**Figure 3:**
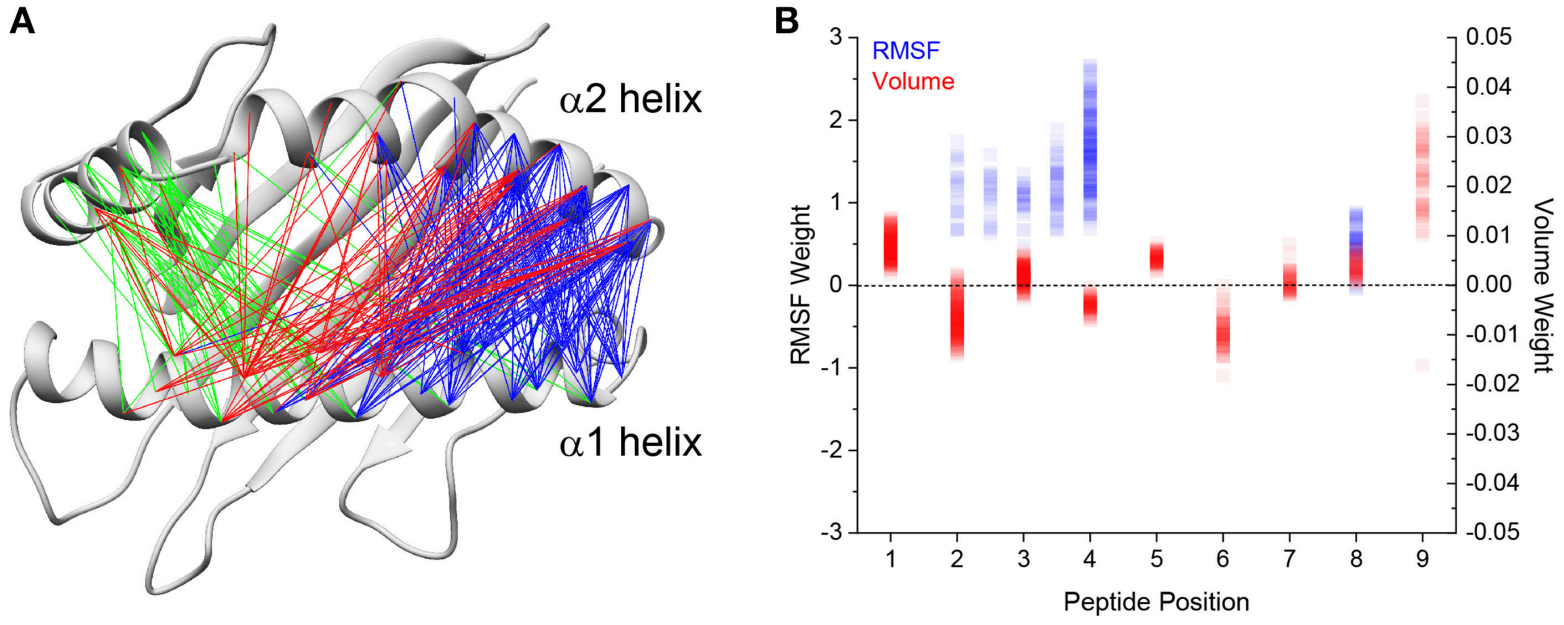
Linear regression models predict fluctuations in binding groove distances. **(A)** Linear regression models were constructed to predict the average α helix Cα-Cα distance in each of the 52 simulations using terms which described physical and chemical differences in the peptides. The final models all have a correlation coefficient >0.6 and were constructed using differences in residue volume and RMS fluctuation. Distances utilizing peptide volume at position 1 through 3 are indicated in blue, peptide volume at positions 4 through 6 indicated in red, and peptide volume at positions 7 through 9 in green. **(B)** Weights of the final models for peptide volume and RMS fluctuations for each peptide position as indicated by the *x* axis. RMS fluctuations at intermediate positions (i.e., 2.5) indicate the averaged RMS fluctuations of those two positions. RMS fluctuations are indicated in blue and volumes indicated in red. Darker colors represent greater sampling at that weight for each term.

We next asked if we could identify peptide features correlated with differential HLA-A2 α helix motion. We previously found a positive correlation between the volume of C-terminal peptide residues and greater fluctuations in peptide binding width (34). Drawing on this finding, we focused on differences in peptide volume at each position of the peptide. We also focused on peptide RMS fluctuations, which we previously showed incorporated chemical features such as side chain charge and hydrophobicity. We constructed multiple linear regression models for each of the 1,102 α1 and α2 pairwise distances described above. For each of the distances, we constructed multiple linear regression models which considered combinations of peptide residue volume and RMS fluctuations as predictors for that distance. In total, we tested ~600,000 different linear models. Following rejection criteria described in the methods, models in which the correlation coefficients were above 0.6 were retained as instances in which the variation in α carbon distance could be reliably predicted. In total, we found that 328 pairs of distances could be well-predicted based on the chosen features. Of these 328, 201 were modulated by volume and motional properties of the N-terminal region of the peptide (positions 1–4; blue lines in Figure 3A), 72 were modulated by properties of the central region of the peptide (positions 4–6; red lines in Figure 3A), and 55 were modulated by the properties of the C-terminal region of the peptide (positions 7–9; green lines in Figure 3A). For the models which utilized the N-terminal region, the modulated distances span regions near the peptide N-terminus to the central region of the peptide binding groove. For the models which utilized the volume at positions 4 through 6, the modulated distances are focused on the middle of the α1 and the α2 helices, yet also stretch from the middle of the α1 helix to the N-terminal end of the α2 helix. For the models which utilized the volume at positions 7 through 9, the modulated distances are primarily those between the short α2-1 helix and the middle of the α1 helix.

For all the models, increases in peptide RMS fluctuation are associated with a broadening of the groove for the predicted distance, indicated by the positive weights given to the RMS terms in the linear models (Figure 3B). In contrast, residue volume results in varied effects depending on the position and model. On average, increases in peptide volume result in an increase in binding groove width for positions 1, 3, 5, 7, 8, and 9. Conversely, increases in peptide volume result in a decrease in binding groove width for positions 2, 4, and 6, possibly due to larger residues strengthening peptide interactions with the HLA-A2 peptide binding groove.

Overall, these results suggest a general mechanism of how different peptides modulate HLA-A2 binding groove dynamics. Increased volumes at or near the peptide termini enhance the breathing of adjacent regions of the α1 and α2 helices, particularly near the N-terminal end of the α1 helix and the short arm and connecting linker region of the α2 helix. This effect is strengthened with peptide fluctuations in these regions, which are also impacted by volume, as well as hydrophobicity as previously demonstrated (34).

### Peptides Alter Protein Fluctuations at Sites Distal From the Peptide Binding Groove

**Figure 4:**
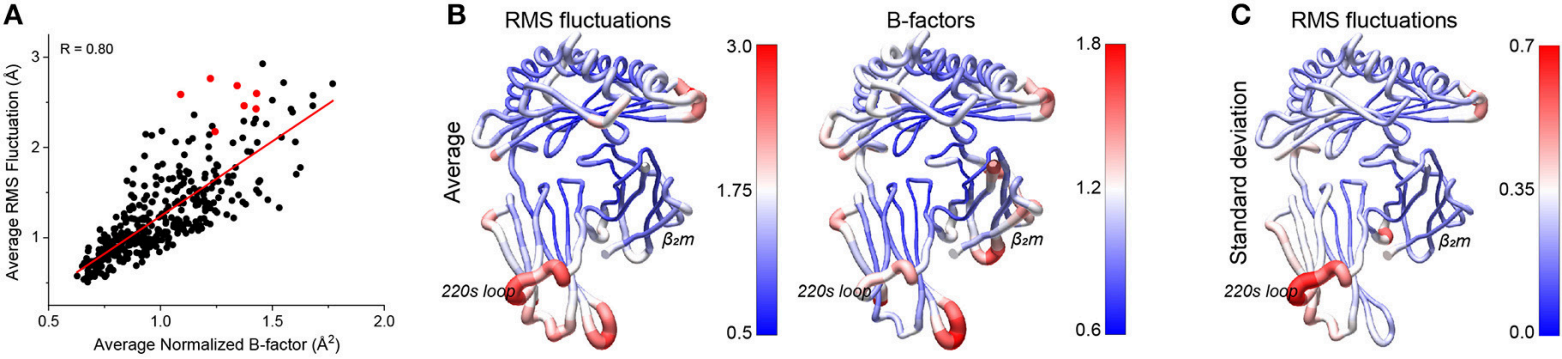
Comparison of peptide-dependent HLA-A2 fluctuations and crystallographic B-factors. **(A)** By-residue average of the Cα RMS fluctuations for the 52 simulations vs. the average normalized crystallographic B-factors from the 52 peptide/HLA-A2 structures. The two sets of data corelate with a coefficient of 0.80. Values for the residues of the 220s loop (amino acids 220-226) are shown in red. **(B)** Average RMS fluctuations from the 52 simulations (left) and average normalized B factors from the 52 structures (right) mapped onto the structure of HLA-A2. **(C)** Standard deviations of the RMS fluctuations from the 52 simulations mapped onto the structure of HLA-A2.

Various experimental and computational studies have shown that peptides can modulate class I MHC protein dynamics at sites other than the peptide binding groove, potentially serving as an indirect signaling mechanism by impacting MHC interactions with NK receptors, coreceptors, and elements of the peptide loading machinery (4, 15, 16, 28–31, 40, 41). We thus asked to what extent our simulations recapitulated these observations. We first compared RMS fluctuations with crystallographic B-factors, which were used recently to identify regions of class I MHC proteins whose motions may be particularly peptide-dependent (40). We compared α carbon RMS fluctuations averaged across all simulations to averaged normalized crystallographic B-factors from the structures used for the simulations. The two datasets were in good agreement, with a correlation coefficient of 0.80 for the entire complex. As seen previously (42), there was a greater spread to the data at higher flexibilities, although the general correlation between high B-factor and high fluctuations held across the entire range (Figure 4A).

Mapped to the structure, the average RMS fluctuations highlighted various regions of the protein which were particularly mobile (Figure 4B). To better distinguish between sites that were simply highly mobile in all simulations vs. those whose mobility was more peptide dependent, we examined the standard deviations of the RMS fluctuations (Figure 4C). Regions of interest that showed high variance included loops of the binding groove near the peptide termini, portions of the β_2_m subunit, and a variety of regions within the α3 domain, including the “220s loop” at the distal end of the molecule. These regions are of note, as the binding groove loops and the α3 domain interact with molecules other than TCR, including NK receptors, coreceptors, and peptide loading chaperones. Additionally, regions of β_2_m have been implicated in communicating the influence of peptide from the groove to other parts of the molecule (29, 43).

As the residues of the 220s loop all exhibited high flexibility but lay above the trendline in Figure 4A, we investigated whether crystallographic contacts could have led to artificially depressed B-factors. Using a cutoff of 5 Å we observed only spurious and inconsistent symmetry related contacts at no greater frequencies than other regions of the protein, suggesting motions here are likely not impacted by crystallographic contacts.

**Figure 5:**
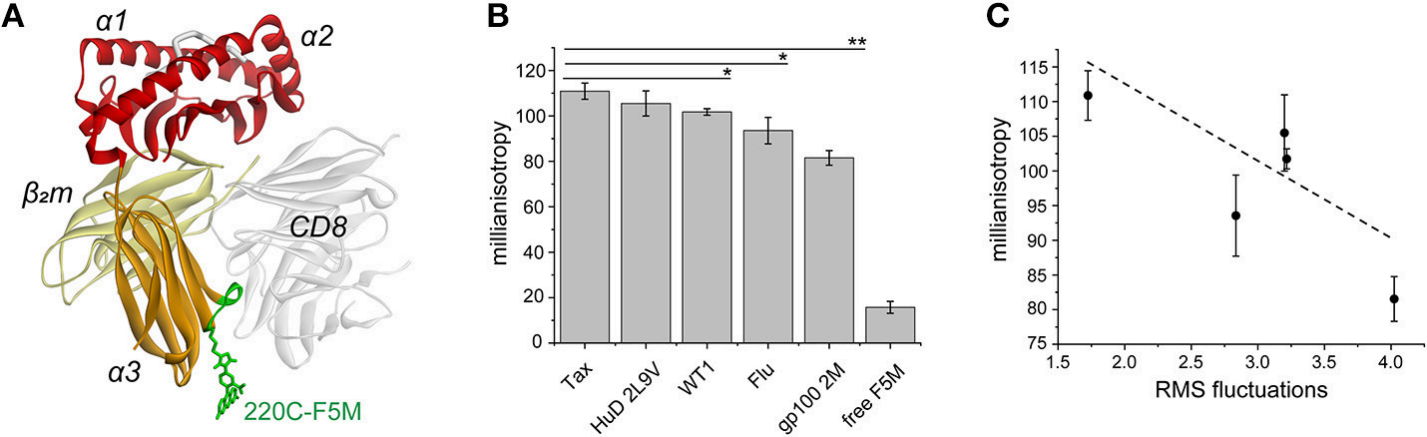
Experimental validation of peptide-modulation of HLA-A2 α3 domain motions via steady state fluorescence anisotropy. **(A)** The HLA-A2 protein and the fluorescent label in the 220s loop of the α3 domain (loop in green; residues 220–226). The binding of the CD8 coreceptor is illustrated to show its relationship to the α3 domain and the 220s loop (44). **(B)** Fluorescence anisotropy (reported in millianisotropy values) measured for D220C-labeled HLA-A2 bound to five different nonameric peptides. For calibration, a fully rigid molecule has a theoretical value of 400, and free fluorescein had a value of < 10. Measurements are the averages and standard deviations from analysis of three independently prepared samples. A single asterisk indicates differences between the Tax sample and the WT1 and Flu M1 samples with *p* < 0.05. The double asterisk indicates a difference between the Tax sample and the gp100_2M_ with *p* < 0.0005. **(C)** Comparison of the measurements for the five peptide/HLA-A2 samples in panel A with the RMS fluctuations at position 220 from the molecular dynamics simulations.

Dynamic communication from the class I MHC peptide binding groove to the α3 domain has been demonstrated experimentally, most recently using hydrogen/deuterium exchange, in which peptide-loaded and peptide-receptive molecules displayed altered exchange behavior in peptide fragments adjacent to a polymorphic site in the 220s loop (Gln224 in HLA-A2) (30). Given the long distance between the binding groove and the 220s loop (~55 Å) we sought to further experimentally assess the extent of intra-protein dynamic communication and how this varies with peptide. We replaced Asp220 near the apex of the 220s loop of the HLA-A2 α3 domain with cysteine, and generated peptide/HLA-A2 samples (Figure 5A). Five tight binding nonameric peptides with ideal primary anchor residues were chosen to allow comparison with the simulations and help ensure that peptide dissociation did not impact the experimental results. We labeled the free cysteine with fluorescein-5-maleimide and measured nanosecond dynamics using steady state fluorescence anisotropy. After removing excess label, we observed small but statistically significant peptide-dependent differences between the measurements with our reference Tax peptide and three peptides (the Wilm's tumor 1 antigen, the influenza M1 antigen, and an anchor-modified variant of the gp100 melanoma antigen) (Figure 5B). Consistent with our selection of tight binding peptides, incubation of samples with excess peptide did not alter the results (e.g., Tax samples without excess peptide yielded an average value of 117 mA; separately prepared Tax samples maintained in 100-fold excess peptide yielded an average value of 116 mA). The anisotropy values correlated with the computed RMS fluctuations at this site (Figure 5C). These results are consistent with different peptides impacting fluctuations in the α3 domain on the nanosecond timescale.

### Pathways of Motion From the Binding Groove to the α3 Domain

**Figure 6:**
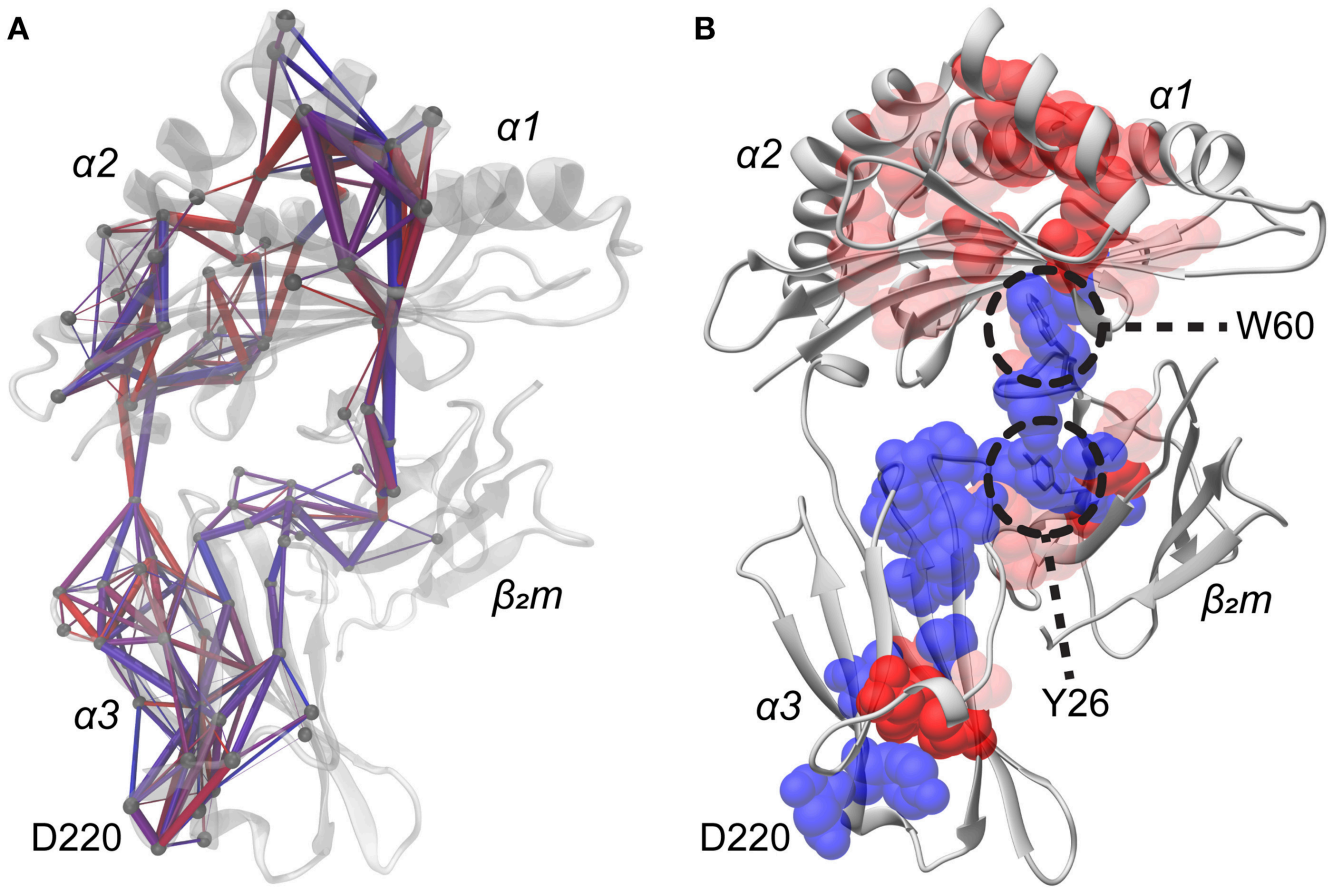
Pathways of covariant side chain dynamics from the peptide to Asp220. **(A)** Representative structure displaying 500 computed pathways from each residue of the peptide to Asp220. Pathways were calculated from a normalized covariance matrix of side chain dynamics. The matrix was filtered to only include those values in which the normalized covariance was >0.4 and if the average Cα distance between pairs of residues was < 12 Å. Paths which have a higher percent utilization are in red with thicker rods, whereas paths which have a lower utilization are in blue with thinner rods. Spheres show Cα atoms of participating residues. **(B)** As in panel A, but composited pathway information from all 52 simulations, highlighting residues which consistently propagate covariant side chain dynamics from the peptide to Asp220 among all 52 simulations. Lists of pathways were composited on a by residue basis for each residue of the peptide. Residues utilized in all nine of these datasets are indicated in blue, whereas residues utilized in fewer datasets are indicated in red, with increasing transparency indicating less frequent usage. Trp60 and Tyr26 are indicated as they were found to be structurally important bridge residues which propagate dynamics across the domains of the protein.

To explore how different peptides can allosterically alter HLA-A2 protein fluctuations at sites remote from the peptide binding groove, we used our simulation data to perform suboptimal pathway analysis between the peptide and the 220s loop (45–47). We calculated normalized covariance matrices for the side chain dynamics for every residue in each of the 52 simulations, focusing on side chains to limit bias from regular backbone secondary structure. Each normalized covariance matrix was filtered to only include values of 0.4 or greater in order to select pairs of residues which were at least moderately correlated, as previously performed (48). Further, correlations were only included if the average Cα distance was within 12 Å in order to exclude covariance resulting from global domain movements. For each simulation, up to 500 pathways were calculated between Asp220 and each residue of peptide, yielding a maximum of 4,500 total pathways per simulation. These pathways were dispersed across the HLA-A2 molecule (Figure 6A), consistent with the notion that altering the protein's energy landscape with different peptides has consequences for global protein motion.

The various pathways identified from each simulation were compared to identify regularly participating, or shared, pathway residues. Residues were considered shared if they were utilized in at least 40% of the multiple pathways found in each simulation. This cutoff was selected to identify residues which were at least moderately conserved in propagating covariant dynamics, yielding a list of residues which displayed high utilization in individual simulations for propagating covariant dynamics from peptide residues to Asp220. From this list we then identified those conserved across multiple simulations. This led us to identify 49 residues with moderate to high conservation across multiple simulations responsible for propagating correlated dynamics from the peptide to Asp220 of the α3 domain. Mapped to the structure (Figure 6B), the list of residues originates from the peptide and connects to most of the neighboring residues in the short arm of the α2 helix, as well as residues of the peptide binding groove floor, notably Ala117, Tyr123, and Ala125. The residues in the binding floor connect to the DE loop of β_2_m and travel through the B strand of the β_2_m protein, ultimately connecting to the α3 domain, the 220s loop, and converging on Asp220.

We examined these conserved residues in detail to determine key sites for propagating dynamics from the peptide to the 220s loop. The most stand-out amino acid was Trp60 of β_2_m. Trp60 lies at the apex of the β_2_m DE loop and interacts with Ala117 in the floor of the peptide binding groove (Figure 6B). NMR studies have previously identified the Trp60 side chain as sensitive to subtle perturbations caused by different peptides bound to the same class I MHC protein (29). A second stand-out residue was Tyr26 of β_2_m, which lies in the interface between the β_2_m B strand and the α3 domain.

To examine the predicted roles of Trp60 and Tyr26 in the pathway of covariant dynamics, we performed new simulations in which either Tyr26 or Trp60 was mutated to alanine and the pathway analysis repeated. Four peptide/HLA-A2 structures were chosen for these simulations, comprising the two that showed the minimum and maximum fluctuations at Asp220 (3MRK [high] and 1DUZ [low]) as well as two that showed intermediate Asp220 fluctuations (3H7B and 3TO2), for a total of eight new 1 μs simulations. Although the proteins remained stable during the simulations, when performing suboptimal pathway analysis no pathways could be identified from the peptide to Asp220 in the α3 domain for seven of the eight simulations. In the remaining simulation (3H7B with the Y26A mutation), the pathways were only partially conserved, either traversing the peptide binding groove and bypassing β_2_m to reach the α3 domain, or bypassing Trp60 by jumping from the peptide to β_2_m via the α1 helix and then traversing the conserved pathway described above. Thus, in all but one case, *in silico* mutation of the key residues eliminated the most conserved connectivity between the peptide and the tip of the α3 domain. We attempted to experimentally validate these observations beyond what has been shown by NMR (29), but consistent with previous reports (49), we found that mutations in the β_2_m-heavy chain interface impaired class I MHC protein stability (as this defect manifests as weaker β_2_m-heavy chain association, we would not expect it to observe it in traditional molecular dynamics simulations of the pre-formed complex; indeed, in either wild-type or mutant simulations we observed no dissociation of β_2_m from the heavy chain).

We next sought potential correlations between peptide properties and fluctuations at the tip of the α3 domain. We performed multiple linear regressions of RMS fluctuations of various peptide positions and those of Asp220. We also considered linear energies between the peptide and residues of the peptide binding groove (50). These terms included counts, averages, standard deviations, and median values of electrostatic and van der Waals interaction energies from the peptide to any residue of the peptide binding groove, resulting in a total of 1,026 terms. Key terms that emerged from the analysis were peptide fluctuations at positions 8 and 9, as well as peptide van der Waals or electrostatic interactions with helical positions 69, 144, and 147. Notably, the latter two amino acids are in the short α2-1 helix and lie adjacent to the peptide the C-terminal end. These observations further implicate the C-terminal half of the peptide and the α2-1 helix in influencing peptide-dependent class I MHC dynamics. We also found though that energetic interactions between the peptide and 23 other HLA-A2 residues could be incorporated into models with statistical significance (p ≤ 0.05). Linear models generated with the four terms mentioned above as well as with any of these 23 interaction terms yield final models with correlation coefficients ranging from 0.79 to 0.81. Thus, although peptide fluctuations near and interactions with the α2-1 helix are particularly important in communicating correlated dynamics through the protein (average *p* < 0.0001), distributed interactions with numerous residues throughout the groove also play a role. As with α1/α2 helix motions, we could not identify correlations between peptide binding affinity and motions in the 220s loop.

## Discussion

Crystallographic structures of peptides presented by class I MHC proteins have provided considerable insight into immune recognition and function. While much work has focused on how peptides bind and how TCRs recognize the resulting composite peptide/MHC surfaces, it is increasingly recognized that rather than serving as a static platform for peptide presentation and molecular recognition, the properties of class I MHC proteins are tuned by different peptides in functionally significant ways. However, the mechanisms by which different peptides exert their effects are not well-understood. Structural studies have typically revealed, at most, minor conformational adjustments to class I MHC proteins with different bound peptides (2, 3, 6, 25, 40, 51, 52). Consistent with these small conformational changes, evidence is now mounting that one way peptides alter class I MHC proteins is by modulating motional properties, both within the peptide binding groove as well as in more distal parts of the protein [as reviewed in (7, 36, 40, 53)]. This concept, sometimes referred to as dynamically driven allostery, is now recognized as a fundamental mechanism of biological regulation (32, 33).

We explored here the peptide-dependent tuning of class I MHC motional properties using molecular dynamics simulations, relying on a large library of lengthy simulations of the class I MHC protein HLA-A2 bound to different peptides. From 52 independent simulations, we observed substantial peptide-dependent effects, and postulated mechanisms for how peptides tune the motion not only of the TCR-facing α helices that form the peptide-binding groove, but also more distant regions of the molecule, including the non-covalently bound β_2_m subunit and the distal α3 domain.

In assessing peptide-mediated effects on the dynamics of the peptide binding groove, we observed a large range of fluctuations, particularly at the N- and C- termini of each helix. The greatest impact was seen at the short arm of the α2 helix (also referred to as the α2-1 helix, adjacent to the C-terminal part of the peptide). Peptide-dependent motions here contributed to large fluctuations in the width of the HLA-A2 binding groove. α2-1 helix structural or dynamic changes have been regularly implicated in peptide-dependent immune functions. For example, different peptides influence the propensity for the α2-1 helix to structurally adapt to incoming TCRs, either favorably or unfavorably influencing TCR binding and resulting in what we have termed an “extension of antigenicity” from the peptide to the MHC (2–6). Adaptations of the α2-1 helix are also believed to be key in promoting peptide exchange by tapasin and the TAPBPR chaperones (9, 10, 18–22, 54). Peptide binding and release from class I MHC proteins is also thought to incorporate motions in this region (25, 30, 36, 55, 56). The motional sensitivity of the α2-1 helix arm thus appears to be a key component of class I MHC biology.

A particularly intriguing observation is the degree to which peptides influence class I MHC motions at regions other than the peptide binding groove, including the α3 domain and the β_2_m subunit. The observation of globally tuned class I MHC protein dynamics has functional implications. For example, peptide-dependent motions can explain the intriguing peptide-sensitivity of inhibitory NK receptors that do not contact the class I MHC peptide binding groove (15, 16). Components of the peptide loading machinery interact not only with the α2-1 helix, but also the α3 domain, and recent data indicates the tip of the α3 domain dynamically responds to peptides (28, 30, 57). A peptide dependence to these interactions, as opposed to a simpler sensitivity to the presence or absence of peptide, suggests the existence of finer control mechanisms influencing immunogenicity and peptide processing and selection than currently recognized.

Our analysis suggests that although communication of correlated dynamics throughout the protein can occur via multiple pathways, a small number of amino acids are preferentially utilized. Two standouts were found in the interface between the base of the peptide binding groove and β_2_m (Trp60), and in the interface between β_2_m and the α3 domain (Tyr26). Both Trp60 and Tyr26 are highly conserved across species and form similar interactions in different class I MHC structures (58, 59). The sensitivity of β_2_m to peptide, and its role in communicating correlated dynamics throughout the protein, can explain findings of enhanced β_2_m association when motions within the peptide domain are restricted via a disulfide linker (60). Consequently, the β_2_m molecule is implicated in sensing and communicating information from the peptide binding groove, elevating its importance above an architectural subunit necessary for class I MHC complex stability.

Our findings support additional roles for polymorphic MHC residues beyond directing peptide selection and influencing TCR binding, as the communication of peptide-dependent dynamics throughout and from the binding groove will be impacted by amino acid composition. Indeed, polymorphisms within the groove have been shown to influence peptide dynamics, as well as motions in the α3 domain (29, 41, 55, 61, 62). Modulation of the motional sensitivity of class I MHC proteins to peptides through polymorphisms suggests roles for class I MHC diversity beyond what is typically considered (i.e., influences on peptide selection and TCR binding).

Lastly, it is likely that the dynamics and thus functions of antigen presenting proteins other than class I MHC proteins are similarly tuned by peptides. For example, the motion and geometry of the binding groove in class II MHC proteins has been shown to be peptide-dependent (40, 63, 64), and comparisons between peptide-loaded, sub-optimally loaded, and empty class II MHC proteins revealed peptide-dependent fluctuations that influence the stability and chaperone-receptiveness of the protein (65–68). Furthermore, changes in class II MHC-presented peptides induce conformational alterations not only in the binding groove (69–72), but also in distal regions of the protein (63, 72), suggesting the existence of similar through-protein, dynamic allostery in class II proteins as we found in class I MHC proteins.

In conclusion, our results provide new insights into the molecular principles governing peptide-dependent effects on MHC proteins. The data indicates that peptide-dependent impacts not only include but extend well-beyond static changes at the peptide-binding groove surface and suggest that MHC proteins in general are highly tunable sensor proteins primed to modulate immunobiology in sophisticated, dynamic fashions.

## Materials and Methods

### Molecular Dynamics Simulations and Analysis

The majority of the molecular dynamics data analyzed here were described previous previously (34). Additional simulations unique to this report were performed identically. Briefly, all simulations were generated with the GPU-accelerated version of the AMBER 14 molecular dynamics suite utilizing the ff14SB force field (73–75). Starting coordinates for each simulation were obtained from the Protein Data Bank; when multiple molecules were present in the asymmetric unit, coordinates from the first were used. Terminal residues of the peptide were modeled in their charged state. Missing side chains and residues, usually localized to the α3 domain of the heavy chain, were modeled in using the crystal structure of HLA-A^*^0201 presenting the Tax peptide (PDB accession code 1DUZ) (39) via Chimera (76). All systems were charge neutralized with sodium counter ions and explicitly solvated with an isometric box of SPC/E water (77) to a minimum of 10 Å from peptide/MHC atoms. Following this, each system was energy-minimized then heated to 300 K using a Langevin thermostat and solute restraints. Following minimization and heating, solute restraints were gradually relaxed from 25 to 0 kcal mol^−1^ Å^−2^ in the NPT ensemble. Volume was then fixed at the average volume of a 100 ps NPT simulation with no restraints. Following a brief 50 ps simulation in the NVT ensemble, production trajectories were then calculated for each system. Production trajectories were calculated in the NVT ensemble with a 2 fs time step. Short range non-covalent interactions were calculated with a 10 Å cutoff, and long-range electrostatic interactions were treated via particle mesh Ewald (78). All bonds involving hydrogen were constrained with the SHAKE algorithm (79). Trajectories were calculated for a total simulation time of 1 μs, with data output every picosecond. Initial velocities for each trajectory were assigned from Maxwellian distribution at the starting temperature utilizing a random seed generated by the date and time.

### Multiple Linear Regression Models Describing Peptide Modulation of the Binding Groove

Multiple linear regression models that related peptide features to HLA-A2 binding groove dynamics were constructed in MATLAB. Terms examined included total residue volume and Cα RMS fluctuations for individual peptide residues and averages of consecutive pairs of residues, as reported previously in our analysis of peptide motions (34). Models were constructed to predict every average pairwise distance between the 29 and 38 α carbons of the α1 and α2 helices, respectively, for a total of 1,102 distances for each simulation. Distances were calculated with the “distance” functionality of cpptraj in the AMBER suite (80). Models for each distance were constructed by considering every permutable combination of peptide residue volumes, up to a total of four individual volume terms, as well as a single peptide RMS fluctuation term (with the RMS fluctuation term reflecting Cα RMS fluctuation for a single residue, or the average of Cα RMS fluctuations for adjacent residues) (34). In total, 613,814 models were constructed, and the correlation coefficient of each model determined. For simplification, these models were pruned to only include those which incorporated consecutive peptide positions. For example, a model in which the volumes of peptide positions 2 through 6 were incorporated was accepted, however a model in which the volumes of positions 1 through 2 and 4 through 5 was not. Further, only models in which the fluctuation term was within or adjacent to the peptide volume range were allowed. This process reduced the total number of models to 336,110, for a total of 305 potential models for each Cα distance. Correlation coefficients for each of these models were extracted and compared for each individual Cα distance in order to identify the best model for each. We found that increasing the number of volume terms for each distance from 3 to 4 only increased the correlation coefficient for that distance by an average of 0.01. Accordingly, final models were limited to those which only incorporated volumes for three residues. Thus, all final models constructed followed the general equation:

$$\begin{array}{l} Distance\;=\;Model.Constant+\left(Vol.\;Weight1\right)Volume1\\ \;+\;\left(Vol.\;Weight2\right)Volume2+\left(Vol.\;Weight3\right)Volume3\\ \;+\;\left(RMSF.\;Weight\right)RMSF \end{array}$$

Following this, the best performing three-volume/single RMS fluctuation model was extracted for each distance, and models for which the correlation coefficient was < 0.6 were excluded from further analysis, resulting in 346 identified distances. Of the remaining distances, we investigated the terms which comprised the best models. In total, 298 of the 346 distances were constructed from one of eight models which were composed of the same terms with different weights. To further reduce complexity, we reduced the permitted combination of peptide volumes and RMS fluctuations (referred to as templates) from 26 to 7. These seven were chosen given that 20 or more distances utilized that particular template. In one instance, we gave preference to a single template over the other as the RMS fluctuation term was within the range of residues in which the volumes were incorporated vs. adjacent. The analysis was performed again while only considering one of the 7 templates for each distance. The correlation coefficient for each distance was extracted for each template, and only the best model for each distance was retained, once again excluding those distances in which the best performing correlation coefficient was < 0.6.

### Suboptimal Pathway Analysis

Pathways were determined as described previously (45–47). Prior to suboptimal pathway analysis, dynamical cross-correlation matrices were calculated for all 52 simulations via the “matrix correl” function in cpptraj for the side chain atoms from each residue to every other residue in every HLA-A2 simulation (using HA2 and HA3 for Gly) following global Cα superimposition to the initial crystallographic coordinates. Values were averaged for each residue from all 52 matrices, resulting in 52 by-residue side chain average dynamical cross-correlation matrices. Using R, these matrices were further processed, omitting values below 0.4 to identify pairs of residues which were at minimum moderately correlated. Further, values were omitted if the Cα atoms of those pairs of residues did not lie within an average of 12 Å throughout the simulation. From this, for each matrix, up to 500 paths of correlated motion were calculated from each residue of the peptide to Asp220 via the “cnapath” function of Bio3D, resulting in a total of 4,500 identified pathways for each simulation partitioned into nine datasets of 500 pathways. We observed that pathways were more frequently present from the peptide C-terminus than any other position (39/52 had no pathways for P1 and P2, 25/52 had no pathways for P3, 35/52 had no pathways for P4 and P5, 27/52 had no pathways for P6, 19/52 had no pathways for P7, 18/52 had no pathways for P8, and 2/52 had no pathways for P9).

For those cases in which pathways from a peptide residue to Asp220 were identified, output for each 500-pathway dataset included residues involved in the identified paths, as well as the percentage of those paths in which those residues were utilized. The output of each 500 pathway dataset was further processed to only include residues in which that particular residue was utilized in at least 40% of the identified paths in order to identify those residues which were highly utilized within that simulation. These processed datasets were then compiled on a by residue basis, for a total of 9 datasets composed of pathways for the 52 individual simulations. Following this, counts of each residue were tallied for each of the 9 datasets to identify those which were not only highly utilized in each simulation via the 40% criterion, but also had a conserved utilization across multiple simulations. A particular residue was considered to have conserved utilization across simulations within a single dataset if the count for that residue was greater than or equal to 10% of the total number of successfully identified pathways for that particular dataset. For example, a residue had to be identified in two simulations in the P1 dataset given that only 13 simulations had identified pathways but had to be identified in 5 simulations in the P9 dataset given that 50 simulations had identified pathways. The conserved residues for each of the 9 datasets were then compiled to identify those which were even further conserved across the 9 datasets, omitting those residues which were only identified in 1 of the 9 datasets. Counts of each residue across the 9 datasets were calculated and then subsequently mapped onto the reference Tax/HLA-A2 structure.

### Protein Expression and Purification

Recombinant HLA-A2 heavy chain and β_2_m were expressed as inclusion bodies in *Escherichia coli* and denatured in 8 M urea. The D220C mutation was made via site-directed mutagenesis and confirmed by sequencing. Synthetic peptides were purchased from AAPPTec. Each peptide/HLA-A2 complex was refolded and purified following established procedures (81). Briefly, inclusion bodies were diluted at a 1:1 ratio in the presence of excess peptide in refolding buffer (100 mM Tris (pH 8), 400 mM L-arginine, 2 mM EDTA, 6.3 mM cysteamine, 3.7 mM cystamine, 0.2 mM PMSF). Complexes were incubated at 4 °C for 24 h. Solutions were then desalted by dialysis against water at room temperature for 48 h. Protein was then purified by anion exchange followed by size-exclusion chromatography. Protein concentrations were determined by measuring absorbance at 280 nm.

### Fluorescence Anisotropy Measurements

For labeling, purified peptide/HLA-A2 complexes were combined with 10-fold excess fluorescein-5-maleimide and 20 μM TCEP-HCl in 10 mM HEPES, 150 mM NaCl (pH 8.3). Labeling reactions were allowed to continue for in the dark for 2 h at room temperature. Samples were then dialyzed for 18 h in the dark at room temperature against 20 mM Na_2_HPO_4_, 75 mM NaCl (pH 7.4), then purified by size-exclusion chromatography to remove excess label. Labeling was confirmed through UV visualization of SDS-PAGE gels. Labeling efficiency was determined via the ratio of absorbance at 494 nm and 280 nm and reached 80%. The fluorescence intensity of a wild-type control (Tax/HLA-A2 lacking the free cysteine at position 220) was typically < 5% of the intensity of the labeled, experimental complexes. Steady-state fluorescence anisotropy experiments were performed on a Beacon 2,000 instrument. Measurements were performed at 25°C with protein concentrations between 50 and 100 nM, averaging at least 50 readings after samples attained thermal equilibrium. Measurements were performed with three independently prepared peptide/HLA-A2 samples. Values are the averages and reported errors are the standard deviations of the three measurements. Control experiments with excess peptide used freshly prepared samples with 100-fold excess peptide added immediately after removal of excess label.

## Author Contributions

CMA and AB performed and analyzed molecular dynamics simulations. CMA, EA, AB, SC, FN, and TE helped interpret simulation data and provided feedback on overall results and directions. CA and LH performed and analyzed fluorescence anisotropy experiments. CMA, AB, TE, SC, LH, and BB conceived of and contextualized the approach. BB oversaw and directed the project. CMA, EA, AB, LH, TE, SC, and BB wrote and edited the manuscript.

### Conflict of Interest Statement

The authors declare that the research was conducted in the absence of any commercial or financial relationships that could be construed as a potential conflict of interest.
