# Supplementary material for: Dynamically Driven Allostery in MHC Proteins: Peptide-Dependent Tuning of Class I MHC Global Flexibility
Source: Front Immunol. 2019 May 3;10:966. doi: 10.3389/fimmu.2019.00966 (PMC6509175; doi:10.3389/fimmu.2019.00966)
Supplement: Supplementary file 1 [file Data_Sheet_1.PDF]

Table S1. Table of peptide/HLA-A*0201 structures whose simulations were analyzed. See also Table S1 in ref. 34 (*evaluated complexes below are the Training Set in ref. 34*).

Evaluated peptide/HLA-A2 complexes			
PDB	Sequence	PDB	Sequence
1B0G	ALWGFFPVL	3GSO	NLVPMVATV
1DUZ	LLFGYPVYV	3GSQ	NLVPSVATV
1EEZ	ILSALVGIL	3GSR	NLVPVVATV
1HHG	TLTSCNTSV	3GSU	NLVPTVATV
1I1F	FLKEPVHGV	3GSV	NLVPQVATV
1I1Y	YLKEPVHGV	3GSW	NLVPMVAAV
1I7T	ALWGVFPVL	3H7B	MLWGYLQYV
1I7U	ALWGFVPVL	3HPJ	RMFPNAPYL
1S8D	SLANTVATL	3I6G	GLMWLSYFV
1S9W	SLLMWITQC	3KLA	SLLMWITQL
1S9X	SLLMWITQA	3MRB	NLVPMVHTV
1S9Y	SLLMWITQS	3MRE	GLCTLVAML
1T1X	SLYLTVATL	3MRF	GLCPLVAML
1T1Z	ALYNTAAAL	3MRG	CINGVCWTV
1TVB	ITDQVPFSV	3MRK	PLFQVPEPV
1TVH	IMDQVPFSV	3PWJ	LLYGFVNYV
2GTW	LAGIGILTV	3PWL	LGYGFVNYI
2GTZ	ALGIGILTV	3PWN	LLYGFVNYI
2V2X	SLFNTVATL	3QFD	AAGIGILTV
2VLL	GILGFVFTL	3TO2	LACFVLAAV
2X4O	KLTPLCVTL	3V5D	KVAELVHFL
2X4S	AMDSNTLEL	3V5H	KVAEIVHFL
2X4U	ILKEPVHGV	3V5K	KVAELVWFL
3FQT	GLLGSPVRA	4I4W	ILAKFLHRL
3FQW	RVASPTSGV	4K7F	VCWGELMNL
3FT4	VLRDDLLEA	4L29	YLLMWITQV
